# A Binary-Entropy Analysis of the Relationship Between Scoring Structure and Match Outcome in Badminton

**DOI:** 10.3389/fpsyg.2022.799293

**Published:** 2022-03-08

**Authors:** Chih-Chuan Wang

**Affiliations:** Office of Physical Education, National Yang Ming Chiao Tung University, Hsinchu, Taiwan

**Keywords:** notational analysis, data analysis, win rate, racket sports, elite player

## Abstract

This study explores the relationship between the scoring structure and the win or loss of a badminton match, while providing quantitative analytic data using binary entropy to determine the uncertainty of said win or loss. Scoring structure data were collected from the official match records of the top 16 events of the World Badminton Championships from 2006 to 2020 (a total of 10 editions) as collection objects (745 matches and 1,734 sets in all) and were analyzed by means of notational analysis. Our entropy analysis showed that the main factor affecting the certainty of win or loss in men’s singles, men’s doubles and mixed doubles comes from the number of leading points, and in women’s singles and women’s doubles from whether the current point is closer to the match point. Our binary-entropy analysis based on scoring structure showed that, to maintain high uncertainty so that players stay competitive, the scoring points of two sides should differ in less than 5; in addition, the decisive factors for victory strongly depend on gender, also justifying research results of previous studies.

## Introduction

Using data analyses of sports competition information, important indicators in athletes’ competition performance can be objectively evaluated, and such data can serve as an important reference for movement analysis, evaluation of techniques and tactics, and decision-making of future training plans and pre-match drawing-up of tactics (Hong and Tong, 2000; Hughes and Franks, 2004; Dieu et al., 2020). Moreover, by analyzing and comparing the competition data of athletes with other athletes, athletes can be evaluated, talents selected, and professional knowledge in the field of sports competition effectively constructed, which has an important contribution to improving the performance of athletes and the accumulation of sports science knowledge (McGarry et al., 2002).

New badminton competition rules were introduced in 2006, in which the original 15-points system was changed to the 21-points system, not only significantly impacting athletes’ careers, training, and tactics planning, but also reducing the usability and reference value of the competition information and scientific research results analyzed in connection with the old badminton competition system. Therefore, many studies have recently been conducted on match time structure, strokes, footwork, and movement of the new badminton competition system (21-points system) (Laffaye et al., 2015; Abdullahi and Coetzee, 2017; Abián-Vicén et al., 2018; Gómez et al., 2019, 2020; Valldecabres et al., 2020), as well as physiological and psychological characteristic performances (Alcock and Cable, 2009; Phomsoupha and Laffaye, 2015), in order to reconstruct the competition information and data analysis results of the new badminton system.

Nevertheless, different scoring states, in particular, the current score and points being ahead/behind the opponent’s, are indisputably essential to players’ strategies for winning a match, as well as the excitement of the game. Notably, the relationship between players’ scoring coordination and point outcome entropy using the temporal-related variables was investigated in Gómez et al. (2021); also, match outcome prediction using Naive Bayes and Feature Weighting technique has been addressed in Sharma et al. (2021). However, the study of badminton from the perspective of scoring status has not yet been seen in the literature.

The extent to which people get excited about sports is closely related to the uncertainty of who the winner will be. Therefore, probability theory and statistics offer a framework for scientific quantitative studies of sports. In fact, various probabilistic models along with numerical search methods have been developed to investigate the scoring issues of such racket sports as tennis, table tennis, squash, and badminton (Clarke and Norman, 1979; Riddle, 1988; Stewart, 1991; Barnett and Clarke, 2002; Seve and Poizat, 2005; Percy, 2009). For badminton specifically, Percy (2009) utilized tools from combinatorics and probability theory to analyze and compare the likelihood of winning a game in both the old and new game rules. In particular, using a simple conditional probability model and assuming that each team has a constant probability of winning any rally against the other and that the outcomes of all rallies are independent, the authors derived for both sets of rules the corresponding probabilities of winning for all types of matches (men’s single/double, women’s single/double, mixed double). Their results can be explored to assess the extent of excitement offered by both scoring systems, as well as for predicting the outcome of the game.

Entropy, which dates back to the seminal work of Shannon (1948) regarding the ultimate data rate for reliable communication, is undoubtedly the most commonly used measure of uncertainty in all disciplines of science and engineering research. In sports research, entropy has been adopted by Hobbs et al. (2020) to investigate how spatial uncertainty of the basketball over the field can impact “effectiveness,” that is, the likelihood of scoring. By dividing the space into disjointed grids, a Bayesian hierarchical model was then used to establish an explicit connection between spatial entropy and effectiveness, thus justifying that the factor of space plays a potentially important role in studying the basketball scoring system. Entropy was also considered by Baio and Blangiardo (2010) and Taylor et al. (2020) to conduct spatial analysis of football. By dividing the football court into multiple disjointed spatial grids, entropy was used to quantify the uncertainty of plays appearing in each grid, which was then exploited to examine and study the strategies of attack/defense in accordance with the trajectories of players and the football under a probabilistic framework. In the study of badminton, entropy is utilized in Gómez et al. (2021) for dynamic analysis of scoring performance of men’s single matches. To the best of our knowledge, the aforementioned works are currently the only entropy-based sports studies. Given that entropy is the core basic element in quantifying uncertainty, much remains to be investigated in the research area of sports.

In this paper, we leveraged entropy to study the impact of scoring states on the likelihood of winning a badminton game. Our study was based on the scoring records of BWF World Championships (top 16 events from 2006 to 2020). We considered five official match types, namely men’s single, men’s double, women’s single, women’s double, and mixed double. Conditioned on the event that the current score was k points and was m points ahead of the opponent, the probability of winning is first computed for each such *scoring state* (k, m). The collection of scoring states over certain k’s and m’s is formally referred to as the *scoring structure*, a list of evolution of scores en route to victory. Since the outcome of the game can be viewed as a (conditional) Bernoulli random variable, the uncertainty of winning associated with the scoring state (k, m) can be naturally assessed using the binary entropy of a Bernoulli random variable. Unlike existing entropy-based studies of sports (Baio and Blangiardo, 2010; Hobbs et al., 2020; Taylor et al., 2020; Galeano et al., 2021) that were developed under specific probabilistic models, our study is model-free, thus eliminating the need to identify model parameters and, more importantly, being immune to potential model uncertainty and inaccuracy. In addition, the computed binary entropy revealed several interesting insights into the match results associated with various scoring states and the potential strategies players should adopt in order to stand an improved chance for winning the match. At the same time, through the relationship between scoring states and the uncertainty of the outcome of the game, it can be used as a reference for future revision of the system.

## Materials and Methods

### Sample and Materials

In this study, we took the top 16 events of the World Badminton Championships from 2006 to 2020 (a total of 10 editions) as collection objects, excluding five matches with incomplete information (such as abandonment due to injury and abstention), so we ultimately obtained information covering 745 matches and 1,734 sets, including 150 matches (341 sets) of men’s singles, 149 matches (349 sets) of women’s singles, 149 matches (353 sets) of men’s doubles, 149 matches (343 sets) of women’s doubles, and 148 matches (348 sets) of mixed doubles. The scoring structure data came from the data source of official match records provided on the official website of the BWF (tournament software), and three badminton experts were invited to develop the scoring structure record form for badminton match for this study by referencing the record form designed by Wang (2017).

### Parameters

This study primarily explored the impact of the scoring structure (bilateral scoring changes in the badminton match) on the win or loss of the match, that is, the win rate situations with various different points obtained first and leading points. For example, when the score was 15:13, the score obtained first (the current score) was 15 and the leading point was 2, thereby a scoring state (15, 2), and the probability of winning the match for this situation was calculated.

After discussions with experts and scholars, under the current 21-points competition system, when the score obtained first reaches 15, the match enters the important stage plays strive to lead all the way to win the match. That we choose the scoring point 15 as the “crunch moment” is justified by our previous study using the top 16 events of World Badminton Championships, indicating that, before scoring point 15, the scoring pattern is of little relevance to the final match outcome; the impact becomes perceptible once larger than 15 (Wang, 2017). Combined with relevant research results in the past, most victory and defeat difference scores in badminton have fallen between 2 and 7 points, with the biggest lead being between 4 and 7 points (Torres-Luque et al., 2019). Accordingly, under the scoring structure of this study, we started analysis when the score obtained first reached 15 (*k* = 15), while the leading scores (m) ranges from 2, 3, 4, 5, and 6 points or more.

### Procedure

In this study, we used notational analysis (Abdullahi and Coetzee, 2017; Gómez-Ruano et al., 2020) to collect data (focusing on the task of building up the scoring status/structure data sets) and invited four badminton experts (coaches and players) to be observers. Prior to the actual analysis, the items to be analyzed were defined and clarified; then the data of five matches were randomly selected for pre-test. After statistical analysis of the data obtained by the four observers, the inter- and intra-observer reliability analysis was assessed as very good (Kappa: > 0.91; correlation coefficient *r* > 0.98; ICC: > 0.95) (Hopkins, 2000).

During the formal analysis, the four analysts were arranged in separate spaces for data label, and completed analytic records were directly handed to the researcher. Among them, the first and second analysts (Team A) independently annotated 5-year data (2007, 2009, 2010, 2011, and 2013), and the third and fourth analysts (Team B) also independently annotated 5-year data (2014, 2015, 2017, 2018, and 2019). After completing data notation, Team A checked the data annotated by the two analysts of Team B, and Team B checked the data annotated by the two analysts of Team A. If any difference was found in the annotated data, it was submitted and confirmed for the correctness of the data by the four analysts together.

### Why Entropy?

In general, the most intuitive description of a match by the public is usually win or loss, but in this paper, we adopted entropy for analysis. The first reason for doing so was that the win rate diagram could not provide a data display consistent with intuition, usually the higher wonderful degree expressed by larger values was consistent with the intuitive reaction in reading diagrams. However, when discussing the wonderful degree, the most wonderful match is one in which the win rates of the two opponents are about the same, that is, when the win rate is 0.5, the value 0.5 is, however, in the very middle of the win rate diagram, being rather counter-intuitive. Furthermore, when the win rate crosses 0.5 and appears on either side of the straight line (*y* = 0.5), it is not easy to directly judge the wonderful degree associated with a given win rate based on its distance to 0.5, namely, the absolute value | win rate-0.5|. Using the first scoring of 20 points with a 3-point lead and a 4-point lead of women’s singles as an example (Table 1), the win rate with a 3-point lead (equal to 1) can be directly seen to be higher than that with a 4-point lead (equal to 0.933); the gap between the two (1−0.933 = 0.067), on one hand, is almost nil when seen from the win rate diagram, and on the other hand hardly indicative of the true difference between their wonderful degree.

**TABLE 1:** Entropy analysis for women’s singles.

	Win rate	Entropy
m = 3 points	1	0
m = 4 points	0.933	0.353

In Table 1, the win rate is not less than 0.5, because if a player has a leading advantage, naturally he/she has a better chance to ultimately win. When the amount of statistical data is large enough, the probability of the win rate being less than 0.5 becomes lower; the second reason is that the difference in statistical win rate values is not big; in many cases, the difference between two points is only a fraction of zero, and such razor-thin difference might be hardly discernable from the win rate diagram (as demonstrated from Table 1). In the win rate diagram, the same absolute values of | win rate-0.5 | correspond to the same wonderful degree (e.g., the wonderful degree is the same at win rate = 1 and win rate = 0); this also implies that the actual usage range in the win rate diagram is only between 0 and 0.5, a bit small.

**FIGURE 1:**
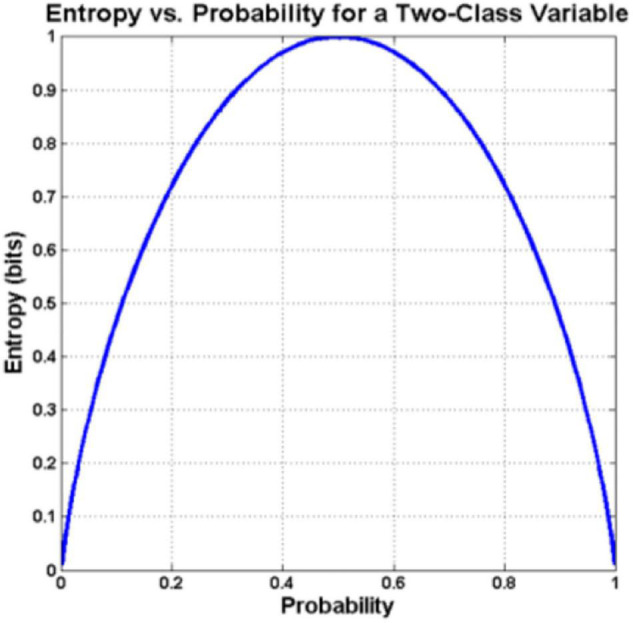
Plot of binary entropy *H(p)* as a function of the parameter *p*.

To eliminate this drawback, in this paper we employed binary entropy, rather than the probability of winning, as a measure of uncertainty (Figure 1). More specifically, for a random variable *X* with n outcomes {x_1_, x_2_,…,x_n_}, the denotation by P(x_i_) is the probability that the outcome x_i_ occurs. Then the entropy of *X* can be as defined by Cover and Thomas (2006).


(1) $$H\,\left(X\right)=-\sum_{i=1}^{n}\text{P}\,\left(x_{i}\right)\,l\,o\,g_{2}\,\text{P}\,\left(x_{i}\right)$$

When specialized to the binary case, i.e., X is a binary random variable with outcomes {x_1_, x_2_}, if we write P(*x*_1_) = *p* = 1−P(*x*_2_), *H*(*X*) in (1) is the binary entropy, which as a function of *p* is reduced to


(2) $$H\,\left(X\right)=-p\,l\,o\,g_{2}\,p-\left(1-p\right)\,l\,o\,g_{2}\,\left(1-p\right)$$

The following figure plots *H*(*X*) in (2) as a function of *p*. We observed from the figure that *H*(*X*) reached its maximum for *p* = 0.5 (i.e., largest uncertainty occurs when the two events are equally likely) and its minimum when *p* = 0 or 1 (i.e., there is no uncertainty when one is definitely sure about which outcome will take place).

**FIGURE 2:**
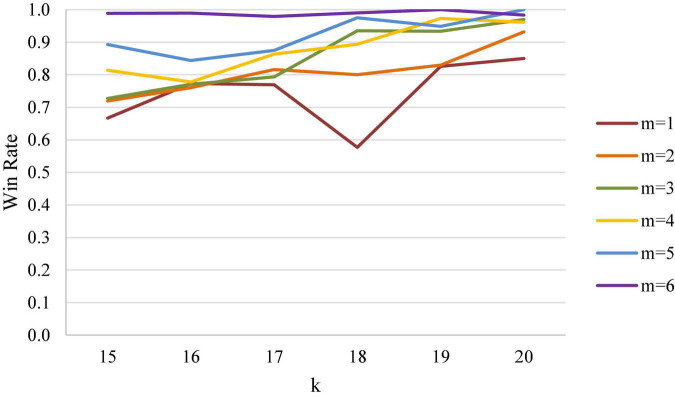
Analysis diagram on win rate of first scoring of 15 points and leading points in women’s singles.

**FIGURE 3:**
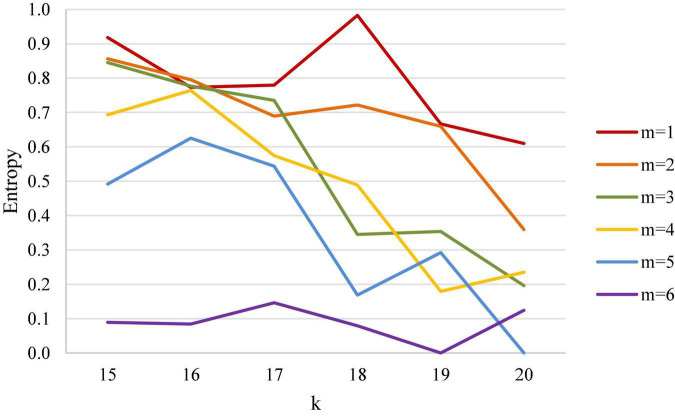
Entropy analysis diagram of first scoring of 15 points and leading points in women’s singles.

Clearly, entropy expands the range from 0–0.5 in the win rate diagram to 0–1, leading to better separation of two distinct points. Using the first scoring of 20 points with a 3-point lead and a 4-point lead of women’s singles as an example, the numerical difference between the two points using entropy in Table 1 was converted into increases from 0.067 to 0.353, demonstrating a bigger gap as compared to the decrease of win rate from 1 to 0.933. As further shown in Figures 2, 3, entropy can better highlight the difference between data; confirming with intuition, high entropy directly corresponds to high wonderful degree and uncertainty, free from the need for additional conversion when working on win rate.

This study examined the first scoring (k) and its current different leading point (m); first scored points were divided into 15, 16, 17, 18, 19, and 20 points, while the leading points were divided into 1–6 points. When a certain leading situation is reached, the leading player wins or loses at the end of the match, and the win rate is converted into entropy for analysis and study.

### Statistical Analysis

We carried out analyses of the data collected in this study with IBM SPSS Statistics for Macintosh, Version 19.0. (IBM Corp. Armonk, New York, NY, United States) and Microsoft Excel 2010. First, the matches and sets of the five individual events competition of badminton and each match outcome (i.e., win, loss) were calculated with descriptive statistics (i.e., frequency). Then Excel was used to calculate entropy according to the binary entropy formula (2).

## Results

### Men’s Singles

**FIGURE 4:**
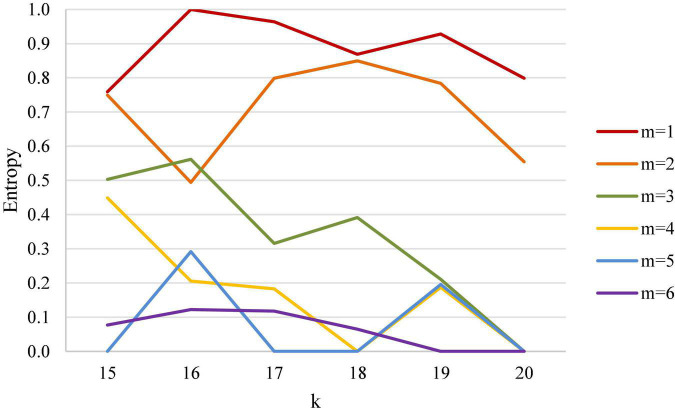
Entropy coefficient line chart for men’s singles.

Based on the actual scoring structure of badminton courts, this study used different scoring states (k, m) (wherein the first scoring was k and the leading point was m) to calculate entropy in order to determine the uncertainty of win or loss of a badminton match. Results of men’s singles analysis are shown in Table 2 and Figure 4. Table 2 demonstrates that in a men’s singles match, when the first scored points k are from 15 to 20 points, entropy decreases with the leading points m. We should first note that, regardless of whether the first scored point being 15 points or even close to the match point, at 20 points, for leading points within 2 points (including 2 points) entropy remains high and does not have a significant decline trend even though first scored point comes close to the match point. We therefore conclude that the uncertainty of the outcome of men’s singles depends mainly on the number of leading points, but less on the first scored point.

**TABLE 2:** Entropy analysis for men’s singles.

Men’s singles	*k* = 20	*k* = 19	*k* = 18	*k* = 17	*k* = 16	*k* = 15
m = 1	0.799	0.928	0.869	0.964	1.000	0.759
m = 2	0.555	0.784	0.850	0.799	0.494	0.750
m = 3	0	0.211	0.391	0.316	0.562	0.503
m = 4	0	0.187	0	0.183	0.206	0.449
m = 5	0	0.196	0	0	0.292	0
m = 6	0	0	0.065	0.118	0.122	0.077

It is noteworthy that when the leading point is greater than 3 points (inclusive) in men’s singles, entropy has a significant downward trend, and the closer the first scored point is to the match point, the more entropy decreases downwards, indicating a higher certainty of the outcome of the match. In other words, when the leading point reaches 3 points (including 3 points) in men’s singles, the closer the point is to match point, and the outcome is almost finalized.

### Men’s Doubles

**FIGURE 5:**
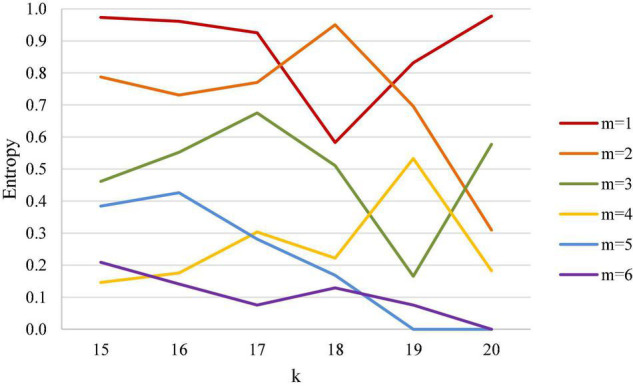
Entropy coefficient line chart for men’s doubles.

The results of men’s doubles analysis are shown in Table 3 and Figure 5. Table 3 demonstrates that in a men’s doubles match, when the first scored points are from 15 to 20 points, entropy declines with the increase of leading points, and the leading point is within 3 points (inclusive). This result shows that, similar to the men’s single case, the uncertainty of men’s doubles match mainly lies in the number of leading points.

**TABLE 3:** Entropy coefficient analysis for men’s doubles.

Men’s doubles	*k* = 20	*k* = 19	*k* = 18	*k* = 17	*k* = 16	*k* = 15
m = 1	0.977	0.831	0.583	0.925	0.961	0.973
m = 2	0.310	0.696	0.950	0.771	0.731	0.787
m = 3	0.577	0.165	0.511	0.675	0.552	0.461
m = 4	0.183	0.533	0.222	0.303	0.176	0.146
m = 5	0	0	0.169	0.281	0.426	0.384
m = 6	0	0.075	0.129	0.076	0.141	0.209

When the leading point is greater than 4 points (inclusive) in men’s doubles, entropy has a significant downward trend, and the closer the first scored point is to the match point, the smaller the entropy coefficient is, which means that when the leading point is greater than 4 points (inclusive) in men’s doubles, the closer the point is to the match point, and the clearer the outcome is.

### Women’s Singles

**FIGURE 6:**
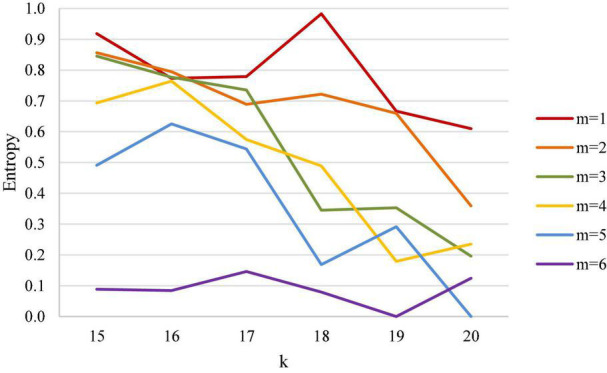
Entropy coefficient line chart for women’s singles.

The results of women’s singles analysis are shown in Table 4 and Figure 6. Table 4 demonstrates that in a women’s singles match, entropy gradually becomes smaller when the first scored point is closer to the match point. When the first scored points are from 15 to 17 points and the leading points are within 5 points (inclusive), the decreasing trend of entropy is not obvious (the outcome is still highly uncertain), and it does not have a decreasing trend even with more leading points. Therefore, we found that, quite different to the men’s single case, the uncertainty affecting the outcome of women’s singles matches is whether the first scored point is closer to the match point.

**TABLE 4:** Entropy coefficient analysis for women’s singles.

Women’s singles	*k* = 20	*k* = 19	*k* = 18	*k* = 17	*k* = 16	*k* = 15
m = 1	0.610	0.667	0.983	0.779	0.773	0.918
m = 2	0.359	0.659	0.722	0.689	0.795	0.856
m = 3	0.196	0.353	0.345	0.736	0.777	0.845
m = 4	0.235	0.179	0.489	0.575	0.764	0.693
m = 5	0	0.292	0.169	0.544	0.625	0.491
m = 6	0.124	0	0.079	0.146	0.084	0.089

It is particularly seen that only after the first scored point is 18 in women’s singles does entropy gradually become smaller with the increase of the leading point, and entropy has a significant downward trend when the leading point is greater than 3 points (inclusive), indicating that after the first scored point reaches 18 points and when the leading point is greater than 3 points (inclusive), the outcome of the match is almost finalized.

### Women’s Doubles

**FIGURE 7:**
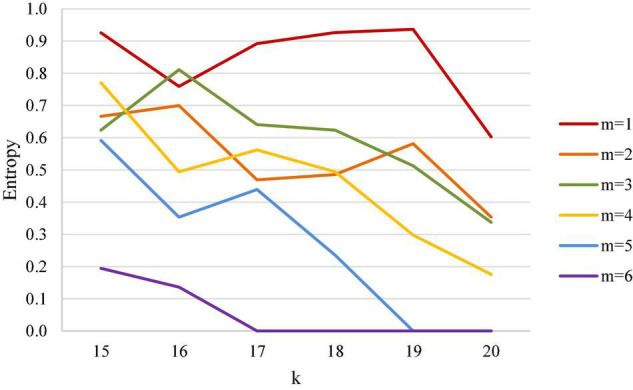
Entropy coefficient line chart for women’s doubles.

The results of women’s doubles analysis are shown in Table 5 and Figure 7. Table 5 demonstrates that in a women’s doubles match, entropy has a sharper downward trend when the first scored point is closer to the match point. When the first scored points are from 15 to 17 points, and the leading points are from 2 to 5 points, entropy does not seem to decline with more leading points; however, after the first scored point is 17 and when the leading point is 2, entropy decreases significantly, and entropy becomes smaller when the first scored point is closer to the match point. In short, after the first scored point reaches 17 points and when the leading point is more than 2 points, the uncertainty of the outcome of the match decreases as the score gets closer to the match point.

**TABLE 5:** Entropy coefficient analysis for women’s doubles.

Women’s doubles	*k* = 20	*k* = 19	*k* = 18	*k* = 17	*k* = 16	*k* = 15
m = 1	0.602	0.937	0.927	0.893	0.759	0.926
m = 2	0.353	0.581	0.485	0.469	0.700	0.667
m = 3	0.337	0.513	0.624	0.641	0.811	0.624
m = 4	0.176	0.297	0.494	0.562	0.494	0.771
m = 5	0	0	0.235	0.439	0.353	0.592
m = 6	0	0	0	0	0.136	0.194

Of particular note, after the first scored point reaches 17 points, the uncertainty of the match has a close relationship with whether the first scored point is closer to the match point and the number of the leading point. If the uncertainty of the result of the match is to be maintained, the relationship between the first scored point and the leading point should have a negative correlation, namely the closer the first scored point is to the match point, the narrower the leading range should be.

### Mixed Doubles

**FIGURE 8:**
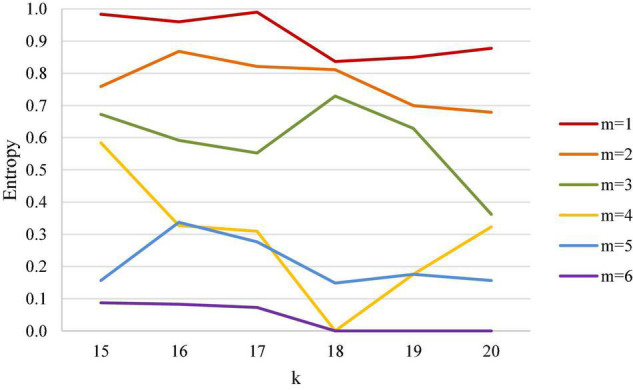
Entropy coefficient line chart for mixed doubles.

The results of mixed doubles analysis are shown in Table 6 and Figure 8. Table 6 demonstrates that in a mixed doubles match, when the first scored points are from 15 to 20 points, entropy declines with the increase of leading points, and when the leading point is greater than 4 (inclusive), entropy has a significant downward trend. This indicates that in the mixed doubles match, when the leading point reaches 4 points (inclusive), the certainty of the outcome of the match is higher. Furthermore, when the leading point is within 3 points (inclusive), regardless of whether the first scored point is close to the match point, entropy does not change much, indicating that the uncertainty of the match is high. Therefore, the uncertainty that affects the outcome of mixed doubles matches comes from the score of leading the opponent.

**TABLE 6:** Entropy coefficient analysis for mixed doubles.

Mixed doubles	*k* = 20	*k* = 19	*k* = 18	*k* = 17	*k* = 16	*k* = 15
m = 1	0.878	0.850	0.837	0.990	0.960	0.983
m = 2	0.679	0.700	0.811	0.821	0.868	0.759
m = 3	0.362	0.629	0.729	0.552	0.592	0.672
m = 4	0.323	0.176	0	0.310	0.327	0.584
m = 5	0.156	0.176	0.149	0.276	0.337	0.156
m = 6	0	0	0	0.073	0.083	0.087

Only when the leading point reaches 4 and 6 points can it show that the first scored point is closer to the match point and that entropy is smaller, further indicating a higher degree of certainty in the outcome of the match.

### Analytic Comparison on the Relationship Between the Leading Points in Five Singles and Entropy

**FIGURE 9:**
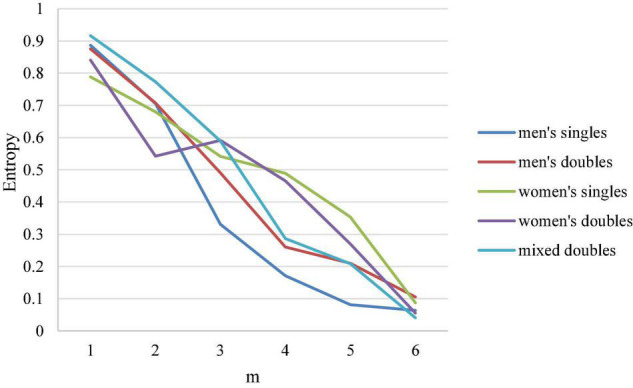
Line chart of different leading points and entropy coefficient.

Figure 9 is a line chart of different leading points in five match types in badminton and entropy coefficient. It is noteworthy from the figure that in women’s events, when the leading points are from 2 to 4 points, the slopes of women’s singles and women’s doubles are −0.095 and −0.012, respectively, while in men’s events, the slopes of men’s singles and men’s doubles are −0.267 and −0.223, respectively, and that of mixed doubles is −0.243, indicating that in comparison with women’s events (women’s singles and women’s doubles), the certainty of the outcome in men’s events and mixed doubles is affected more by the difference in leading points.

## Discussion

To the best of our knowledge, this study is the first to discuss the winning and losing relationship of the five singles in badminton matches from the perspective of scoring structure. Mathematical quantification (using entropy) was adopted for computational analysis to determine the uncertainty of the win or loss of a badminton match. The research results demonstrated the following:

1. In the scoring system of the new badminton competition system, if the high uncertainty of a match is to be maintained, the relationship between the first scored point and the leading point should have a negative correlation, namely the closer the first scored point is to the match point, the narrower the leading range should be. This result is quite consistent with intuition.
2. In men’s singles, men’s doubles, and mixed doubles, whether the first scored point is close to match point or not, the more the leading point is, the lower the entropy coefficient is, indicating that a greater leading point dominates the uncertainty of the match’s outcome.
3. The situations of women’s singles and women’s doubles are just the opposite; as long as the first scored point is closer to the match point, regardless of the score difference between each other, the entropy coefficient is relatively low, indicating that the closer it is to match point, the win or loss of the match is roughly determined.

The aforementioned findings of this study are discussed as follows. First of all, we used entropy in this study to pin down the relationship between different first scored points, leading points, and outcome in the scoring system of the new badminton competition system. We found that the closer the first scored point is to the match point or the more the leading point is, the lower the uncertainty of the match is. This means that in badminton matches, when the score is ahead of the opponent, one should strike while the iron is hot to expand the lead, whereas when the opponent is in the lead, one should narrow the gap to maintain competitiveness in the match (Percy, 2009; Wang, 2017). In terms of entropy, this study puts forward quantitative data to provide more specific advice, namely in all the five match types, when the leading point is 1 point, the uncertainty of the outcome of the match is highest, but when the leading point reaches more than 5 points, the outcome of the match is nearly determined. Therefore, to maintain competitiveness in the match, one should strive to maintain a 5-point gap with the opponent. This result echoes the research of Percy (2009), and, more importantly, it confirms that entropy is an effective measure for characterizing the relationship between victory and defeat in a sports competition system, thus providing an important reference for objective and scientific training and competition system planning.

Secondly, important findings of this study include that in men’s singles, men’s doubles, and mixed doubles, the main factor affecting the certainty of victory or defeat comes from the number of leading points, while in women’s singles and women’s doubles, whether the first scored point is closer to the match point is most influential. In particular, in men’s singles and mixed doubles, when the leading points are more than 3 points (inclusive), and in men’s doubles when the leading points are more than 4 points (inclusive), the outcome of the match is almost finalized. Meanwhile, in women’s singles, after the first scored points reach 18 points and in women’s doubles after the first scored points reach 17 points, the certainty of the outcome of the match is greater. In other words, in women’s singles and women’s doubles, even if the lead is as many as four points or even five points, as long as the first scored points at that time are not more than 17 points, the one lagging behind still has a chance of winning.

We also found in this study that in men’s singles, men’s doubles, and mixed doubles, the certainty affecting the outcome of the match comes from the number of the leading point, while in women’s singles and women’s doubles, the first scored point being closer to the match point has a greater influence. This result echoes previous research results in that different match characteristics will show in the five singles events of badminton, among which the main influencing factors are the different genders of the players and the number of the participants (Abián-Vicén et al., 2013, 2018; Gawin et al., 2015; Valldecabres et al., 2017; Gómez et al., 2019; Torres-Luque et al., 2019). Previous studies have confirmed that male players have higher physiological indicators (such as physical ability and strength) than female players, so male players have higher explosive power and smash frequency and show higher sports intensity performance, of which the proportion of the direct scoring through smash is 29.1% (Abián-Vicén et al., 2013; Gómez et al., 2019; Torres-Luque et al., 2019). Therefore, in men’s events, when the leading point difference is small, male players can actively take points by way of continuous attack to narrow the gap in the score and increase the uncertainty of the match’s outcome. However, the active attack method to take points easily consumes a lot of energy, and when the score gap is too big, changing the score through this way to improve the win rate of the match is not easy. Relatively speaking, because of the lack of the weapon of a finishing shot, female players often need to strike back and forth to create better timing for key points, or to wait for their opponents to make a mistake to take a point. Work density and percentage of time played, average rally (strokes), and average rally(ies) in women’s events are all higher than those of men’s events (Gawin et al., 2015; Gómez et al., 2019; Torres-Luque et al., 2019), showing that it is not easy for female players to take points through the active attack way with fewer strikes in the match, so that a large score gap is dominant factor hard to change in a short period of time. Therefore, the closer the first-scored point of women’s events is to the match point, the higher the certainty of the outcome of the match is.

Through further exploration, we found in this study that the outcome of the match in men’s singles is almost determined when the leading point is more than 3 points (inclusive), but the outcome of the match in men’s doubles is clear only when the lead is more than 4 points, indicating that the uncertainty of the outcome of the match in men’s doubles is higher than that in men’s singles. This result is consistent with previous related research indicating that singles and doubles are mainly affected by the differences in the match court and the number of players, resulting in considerable differences in play and tactics. Men’s singles stress efficient movement about the court and varied shot combinations (clear, drop, smash, lift, or net shot), while men’s doubles focus more on flat shots and attacks, emphasizing faster and more attacks, so that average rally (strokes) and average rally (ies) in men’s doubles are both lower than those in men’s singles (Alcock and Cable, 2009; Gawin et al., 2015; Torres-Luque et al., 2019). Therefore, in men’s doubles, with its emphasis on fast attacks and fast play, the score can easily change (score or loss of points) in short strokes or time, resulting in higher match uncertainty.

In women’s events, we found that the main factor affecting the certainty of outcome in women’s singles and women’s doubles is whether the lead is closer to the match point. The analysis results show that after the first scored point is 18 in women’s singles and when the leading point is greater than 3 points (inclusive), the outcome of the match is almost finalized. Meanwhile, as long as the first scored point is 17 in women’s doubles and the leading point is greater than 2 points (inclusive), the outcome of the match becomes clear. This finding shows that the uncertainty of the outcome of women’s singles is higher than that of women’s doubles; interestingly, this result is the opposite of the men’s events described above. The above relevant studies have demonstrated that the main scoring methods of female players are the organization of back and forth of multiple strikes and opponents’ mistakes. Coupled with the tactical difference between singles and doubles, forced and unforced errors are prone to occur in the process of being mobilized for movement about the court and varied shot combinations in women’s singles. In women’s doubles, two players’ participation in the match can make a front and rear division of the play and rotation to reduce the movement distance, but they lack powerful fast attacks as in men’s doubles, giving priority to taking more shots to organize attacks or defensive attack in women’s doubles. That is why in five match types of badminton events, women’s doubles tend to have higher total shots per match and longest strike strokes and average strike strokes (Abián-Vicén et al., 2018; Gómez et al., 2019; Torres-Luque et al., 2019).

Finally, in mixed doubles, the position mode of female in front and male in rear is a commonly adopted tactic; the female player blocks the net, and the male player attacks in the backcourt. Therefore, in mixed doubles, the male player plays an important and key role in organizing the shot route and launching strikes to take points, so that the scoring structure and competition characteristics in mixed doubles tend to be the same as in men’s doubles rather than in women’s doubles (Torres-Luque et al., 2019).

### Practical Implications

The analysis results of this study confirm that entropy can be used effectively in the field of sports science. As a new method for exploring the uncertainty of the scoring structure change and the outcome of the match, entropy not only expands knowledge in the field of sports, but also makes practical contributions via quantitative analysis. In particular, Percy (2009) pointed out that the excitement and wonderful degree of the match lie in the uncertainty of the outcome of the match. Therefore, the uncertainty of the outcome of a match calculated through entropy to determine the rationality of the new rules can provide more objective and scientific methods, as well as a reference basis, for formulating sports rules or competition systems in the future. In practice, gender differences of players have been confirmed to produce different competition characteristics, and clarifying these differences will help the technical and tactical application and strategy arrangement in both training and matches, as well as highlight the importance of design and simulation of competition characteristics consistent with different genders (Gómez et al., 2019, 2020).

### Limitations and Future Directions

Some factors may limit the scope of this study. First, although this study analyzes and discusses the scoring structure of actual matches, this study makes its exploration only via scoring structure, reasoning of the influencing of the relationship between winning and losing a match therefore being somewhat limited. Notably, reflection of the badminton evolution over years, the variation of players’ performances along seasons, and any other situational variables into our research work definitely call for explicit mathematical models to pinpoint the specific factors to be considered. However, since our study is model-free, it is therefore not clear as how such mathematical models (if in existence) can be incorporated into our analysis and discussions. Despite this, we are aware of the facts that the presented scoring status/structure can be regarded as outcomes summarizing of all the related situational variables. Still, we believe such issues are worthy of our efforts once a model-based framework can be further devised, which is definitely one important future work.

Secondly, from the support of these study results and the results of previous studies (Alcock and Cable, 2009; Gawin et al., 2015; Torres-Luque et al., 2019), we inferred that the main factors influencing the uncertainty of the outcome of the five match types are the difference in the genders of the players and the number of participants, but empirical research support is still lacking. Therefore, we suggest that experimental studies be conducted to further explore this issue in order to provide empirical supporting data. Finally, in terms of future study topics, this study proves that entropy calculation and analysis can be effectively used to explore the scoring structure and the certainty of the outcome of the match. Future studies can apply entropy to the field of sports and provide a platform for further applications of this analytical approach to other aspects of sports events (e.g., table tennis, volleyball, tennis, etc.), as well as players’ movement path or kick-in possession (Taylor et al., 2020) and space and time variables of the sports field, such as the entropy of badminton player’s returning the shot to different positions in the back court (Galeano et al., 2021), from which important match information is obtained to help coaches or players in training and tactical planning during the preparation period and the match period to improve performance in sports matches.

## Conclusion

This study is the first to explain the decisive factors that affect the uncertainty of the outcome of the match via analysis of the scoring structure in badminton matches. Unlike previous studies that focused on the analysis of techniques and tactics, as well as training methods, the analysis of the scoring structure in this study offers a new aspect for understanding the factors that determine the outcome of badminton matches. Through the introduction of the specific scoring structure, the significant differences in the relationship between score evolution and final outcome derived from different “genders” in the “five individual events” of badminton can be more directly reflected. It is worth mentioning that the analysis framework of the scoring structure is not limited by the type of events (singles or doubles) or gender, so more objective and comprehensive analysis and exploration can be made. The results of the analysis, through the calculation of entropy, provide easily observed and quantitative scientific data and verify that the outcome of a match is almost determined when the leading point reaches more than 5 points. The results also confirm that in the five individual events of badminton, competitive events of different genders may influence the scoring structure and the certainty of the outcome of the match (the main factor determining the outcome of the male event is the number of leading points, while in the female event, whether the first scored point is closer to the match point matters). This finding indicates that the differences in genders and physical development of the players need to be stressed in the training and tactical drafting of the badminton players, that different training plans and tactical arrangements should be developed, and in the process of actual competition, more attention should be paid to the score gap with the opponent in male events and mixed events, while in female events, after the score reaches 15 points, the score lead needs to be maintained, thus increasing the possibility of winning.

## Data Availability Statement

The original contributions presented in the study are included in the article/supplementary material, further inquiries can be directed to the corresponding author/s.

## Author Contributions

C-CW developed the project, reviewed the literature, registered the data, and wrote the manuscript.

## Conflict of Interest

The author declares that the research was conducted in the absence of any commercial or financial relationships that could be construed as a potential conflict of interest.

## Publisher’s Note

All claims expressed in this article are solely those of the authors and do not necessarily represent those of their affiliated organizations, or those of the publisher, the editors and the reviewers. Any product that may be evaluated in this article, or claim that may be made by its manufacturer, is not guaranteed or endorsed by the publisher.
